# Predicting lncRNA-disease associations and constructing lncRNA functional similarity network based on the information of miRNA

**DOI:** 10.1038/srep13186

**Published:** 2015-08-17

**Authors:** Xing Chen

**Affiliations:** 1 National Center for Mathematics and Interdisciplinary Sciences, Chinese Academy of Sciences, Beijing, 100190, China; 2 Academy of Mathematics and Systems Science, Chinese Academy of Sciences, Beijing, 100190, China

## Abstract

Accumulating experimental studies have indicated that lncRNAs play important roles in various critical biological process and their alterations and dysregulations have been associated with many important complex diseases. Developing effective computational models to predict potential disease-lncRNA association could benefit not only the understanding of disease mechanism at lncRNA level, but also the detection of disease biomarkers for disease diagnosis, treatment, prognosis and prevention. However, known experimentally confirmed disease-lncRNA associations are still very limited. In this study, a novel model of HyperGeometric distribution for LncRNA-Disease Association inference (HGLDA) was developed to predict lncRNA-disease associations by integrating miRNA-disease associations and lncRNA-miRNA interactions. Although HGLDA didn’t rely on any known disease-lncRNA associations, it still obtained an AUC of 0.7621 in the leave-one-out cross validation. Furthermore, 19 predicted associations for breast cancer, lung cancer, and colorectal cancer were verified by biological experimental studies. Furthermore, the model of LncRNA Functional Similarity Calculation based on the information of MiRNA (LFSCM) was developed to calculate lncRNA functional similarity on a large scale by integrating disease semantic similarity, miRNA-disease associations, and miRNA-lncRNA interactions. It is anticipated that HGLDA and LFSCM could be effective biological tools for biomedical research.

Based on the assumption of the central dogma of molecular biology, genetic information is stored in protein-coding genes and RNA is just an intermediary between a DNA sequence and its encoded protein^1^,^2^. However, sequence analysis shown that there were only **~**20,000 protein-coding genes in the human genome and more than 98% of the human genome does not encode protein sequences^3^,^4^,^5^,^6^,^7^,^8^,^9^,^10^ , yielding tens of thousands of non-coding RNAs (ncRNAs). Based on accumulating experimental evidences, these ncRNAs have played very fundamental and critical roles in various biological processes^11^. Based on whether transcript lengths are larger than 200 nucleotides, ncRNAs can be further divided into small ncRNA (such as miRNA, siRNA, and piRNA) and long ncRNA (lncRNA). Long non-coding RNAs (lncRNAs) are a heterogeneous class of ncRNAs with non-protein-coding transcripts longer than 200 nucleotides^8^,^12^,^13^. In comparison with protein-coding genes, lncRNAs have the following differences: (1) lncRNAs have less conservation across species^14^,^15^; (2) lncRNAs have relatively lower expression level and much more tissue-specific pattern^16^,^17^,^18^. (3) lncRNAs have longer, but fewer, exons. In the early 1990 s, H19 and Xist were first identified based on traditional gene mapping approaches^19^,^20^,^21^. In the recent few years, there has been rapid development in both experimental technology and computational prediction algorithm for lncRNA discovery. Thousands of lncRNAs have been discovered in eukaryotic organisms ranging from nematodes to humans^15^,^16^,^22^,^23^. For example, based on tiling arrays, HOTAIR (HOX antisense intergenic RNA) and HOTTIP (HOXA transcript at the distal tip) were discovered in the homeobox gene regions (HOX clusters)^24^,^25^. Guttman, *et al.*^23^ discovered 1600 novel mouse lncRNAs by integrating gene expression data, the presence of chromatin marks for promoter regions and gene bodies, and the known annotations of coding transcripts. Cabili, *et al.*^14^ generated the human lincRNA catalog across 24 different human cell types and tissues based on chromatin marks and RNA-sequencing (RNA-seq) data.

In recent several years, accumulating experimental studies have shown that lncRNAs play important roles in various critical biological process, such as cell proliferation, differentiation, chromatin remodeling, epigenetic regulation, genomic splicing, transcription, translation and so on^9^,^12^,^18^,^22^,^23^,^26^,^27^,^28^,^29^. Specifically, lncRNA can bind to proteins or miRNAs, resulting in functional inhibition of proteins and titration of miRNAs, respectively^30^. According to the molecular mechanism of lncRNAs, the emerging archetypes of molecular functions of lncRNAs could be divided into signals, decoys, guides, and scaffolds^31^. It has been demonstrated that lncRNA have a very complicated regulation network, but the underlying mechanism of lncRNA-related regulation is still remain unclear. In the light of important biological functions of lncRNAs, the alterations and dysregulations of lncRNAs have been associated with the development and progression of many different complex diseases^12^,^18^,^26^, including cardiovascular diseases^32^, neurological disorders^33^, diabetes^34^, HIV^35^ and various types of cancers, such as breast cancer^36^,^37^, hepatocellular cancer^38^,^39^, prostate cancer^40^,^41^, lung cancer^42^,^43^. In the past few years, many researchers have focused their researches on lncRNA-disease associations, and they have found some specific lncRNAs associated with various diseases. For example, lncRNA HOTAIR has 100 to approximately 2,000 times expression levels in breast cancer metastases based on quantitative PCR^37^,^44^, and its expression level are correlated with metastasis and progression of other various cancers, such as colorectal cancer^45^,^46^, gastric cancer^47^,^48^, liver cancer^49^, lung cancer^47^ and so on. Therefore, HOTAIR was considered as potential biomarker in various types of cancers^45^. Except for HOTAIR, the dysfunction of lncRNA H19 is also involved in various diseases. For example, H19 could be used as a potential prognostic tumour marker for the early recurrence of bladder cancer^50^. Furthermore, it has been demonstrated that down-regulation of H19 significantly decreases breast and lung cancer cell clonogenicity and anchorage-independent growth based on a knockdown approach^36^. Several experimental studies have also shown that lncRNA BCAR4 is associated with breast cancer, which is expressed in 27% of primary breast tumors^51^,^52^,^53^. Specifically, in human ZR-75-1 and MCF7 breast cancer cells, the forced expression of BCAR4 causes cell proliferation in the absence of estrogen and in the presence of various antiestrogens, indicating BCAR4 could considered as a proper target for the treatment of antiestrogen-resistant breast cancer^51^.

Considering the important roles of lncRNAs in various biological processes regulation and complex diseases development and progression, potential disease-lncRNA associations identification could not only benefit the underlying disease mechanism mining at lncRNA level, but also facilitate disease biomarkers detection and drug discovery for disease diagnosis, treatment, prognosis and prevention^29^,^54^. Computational models and tools can effectively decrease the time and cost of biological experiments by quantifying the association probability of each lncRNA-disease pair and verifying most promising lncRNA-disease pairs with high scores based on further biological experimental validation. Nowadays, developing effective computational models by integrating various kinds of biological datasets to prioritize disease-related lncRNAs has become one of the most important and attracting topics in the fields of both lncRNAs and complex diseases.

Some computational models have been developed to infer novel disease-lncRNA associations. In the previous study, Chen *et al.*^54^ presented a semi-supervised learning method, LRLSLDA, to infer novel human lncRNA-disease associations. LRLSLDA was developed based on the assumption that similar diseases tend to interact with functionally similar lncRNAs and the framework of Laplacian Regularized Least Squares. LRLSLDA is a reliable tool for lncRNA-disease association prediction. More importantly, it does not need negative samples. However, the parameter selection problem and the problem of combining two different classifiers into the final classifier exist in this method. Based on the same assumption, Sun *et al.*^55^ presented a method to constructed a lncRNA-lncRNA functional similarity network, then they proposed a global network-based computational method named RWRlncD by integrating disease similarity network, lncRNAs functional network and known lncRNA-disease associations. However, this method can’t be applied to the lncRNAs without any known associated diseases. Li *et al.*^56^ developed a simple genomic location based bioinformatics method for the prediction of novel associations between lncRNAs and vascular disease. However, not all of the lncRNAs are related with their neighbor genes and no statistical tests were used, which resulted in limitations of this method. Yang *et al.*^57^ investigated lncRNA-disease associations by constructing the lncRNA-disease association network and coding-non-coding gene-disease bipartite network based on known associations between diseases and disease genes. Then, a propagation algorithm was applied to infer the underlying lncRNA-disease associations. This method also has some limitations, such as the lack of the information of non-coding genes and protein coding genes interactions and similarities and lncRNA functional annotation. Zhao *et al.*^58^ developed the naive Bayesian classifier to identify cancer-related lncRNAs based on the integration of genome, regulome and transcriptome data. The important limitation of this method is that they regard the unknown lncRNA-disease associations as negative samples, which would largely influence the predictive accuracy of the method. Recently, based on the findings that lncRNAs that sharing significantly enriched interacting miRNAs tend to be associated with similar diseases, Zhou *et al.*^59^ proposed a novel method named RWRHLD to identify candidate lncRNA-disease associations by integrating miRNA-associated lncRNA-lncRNA crosstalk network, disease-disease similarity network, and known lncRNA-disease association network into a heterogeneous network. Then, a random walk was implemented on this heterogeneous network. This method can only predict associations for the lncRNAs that have lncRNA-miRNA interaction datasets, limiting the wide application of RWRHLD. Aforementioned methods all need the prior information of known experimentally verified lncRNA-disease association. So far, although plenty of biological datasets about lncRNA sequence and expression have been generated and stored in some publicly available databases, such as NRED^60^, lncRNAdb^28^, NONCODE^61^, the number of lncRNAs reported to be associated with diseases is still very limited. Liu *et al.*^62^ developed a method by integrating human lncRNA and gene expression profiles, and human disease-associated gene data. This method didn’t rely on known lncRNA-disease associations and obtained an AUC of 0.7645 for non-tissue-specific lincRNAs. However, too many false positives would be brought based on the ROC curve in that paper.

Nowadays, plenty of experimentally confirmed miRNA-disease associations have been collected in various databases^63^,^64^,^65^,^66^. Therefore, the model of HyperGeometric distribution for LncRNA-Disease Association inference (HGLDA) was developed here to predict potential lncRNA-disease associations by integrating known miRNA-disease associations and lncRNA-miRNA interactions. Although HGLDA didn’t rely on any known disease-related lncRNAs associations, it still obtained a reliable AUC of 0.7621 in the leave-one-out cross validation (LOOCV) based on known experimentally verified lncRNA-disease associations from the LncRNADisease database^29^. HGLDA was also applied to predict Breast Cancer, Lung Cancer, and Colorectal Cancer-related lncRNAs. Seven, seven, and five predicted potential associations with false discovery rate (FDR) less than 0.05 have been confirmed by recent biological experiments for these three important human complex diseases, respectively. Above results effectively demonstrated its potential ability of inferring disease-lncRNA associations and detecting biomarkers detection for human disease diagnosis, treatment, prognosis and prevention. Furthermore, the model of LncRNA Functional Similarity Calculation based on the information of MiRNA (LFSCM) was developed to quantitatively calculate lncRNA functional similarity on a large scale by integrating disease semantic similarity, miRNA-disease associations, and miRNA-lncRNA interactions.

## Results

### Performance evaluation of potential lncRNA-disease association prediction

**Figure 1:**
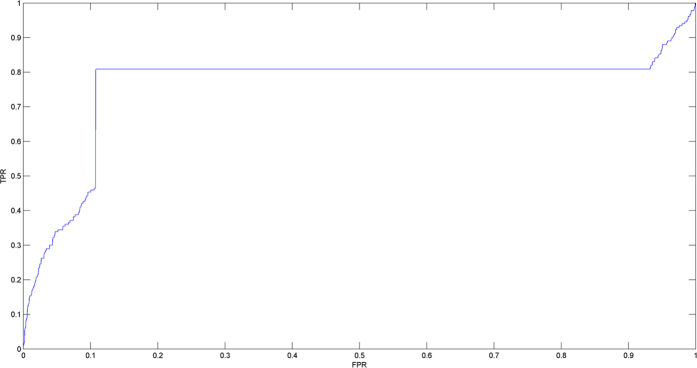
Performance evaluation for the HGLDA in terms of ROC curve and AUC based on LOOCV. As a result, HGLDA achieved an AUC of 0.7621, demonstrating its reliable predictive ability even if potential lncRNA-disease associations were predicted without relying on the information of known disease-lncRNA associations in the model of HGLDA.

HGLDA was applied to the known experimentally verified lncRNA-disease associations in the lncRNADisease database in the framework of LOOCV. Each known disease-lncRNA association was left out in turn as test sample. How well this test sample was ranked relative to the candidate samples (all the disease-lncRNA pairs without the evidence to confirm their association) was evaluated. When the rank of this test sample exceeds the given threshold, this model was considered to provide a successful prediction. When the thresholds were varied, true positive rate (TPR, sensitivity) and false positive rate (FPR, 1-specificity) could be obtained. Here, sensitivity refers to the percentage of the test samples whose ranking is higher than the given threshold. Specificity refers to the percentage of samples that are below the threshold. Receiver-operating characteristics (ROC) curve was drawn by plotting TPR versus FPR at different thresholds. Area under ROC curve (AUC) was further calculated to evaluate the performance of HGLDA. AUC = 1 indicates perfect performance and AUC = 0.5 indicates random performance. As a result, HGLDA achieved an AUC of 0.7621 (see Fig. 1). One important fact must be pointed out is that HGLDA predict potential lncRNA-disease association without relying on the information of known disease-lncRNA associations. Although previous study of predicting potential lncRNA-disease associations by integrating disease-gene associations and gene-lncRNA co-expression relationship obtained a comparable AUC of 0.7645, the ROC curve in that study is much below the ROC curve in this study when FPR is small, which is particularly important for practical biological research. More importantly, available experimentally verified disease-miRNA associations are still comparatively rare relative to the known disease-gene associations. The performance of HGLDA would be further improved when more known miRNA-disease associations could be obtained in the future.

### Case studies of potential lncRNA-disease association prediction

HGLDA was applied to predict potential disease-lncRNA associations for all the diseases investigated in this article. Potential predictive associations with significant FDR values were publicly released to benefit the biological experimental validation (see Supplementary Table 1). It is anticipated that these potential lncRNA-disease associations which significantly share common miRNAs could be validated by biological experiments and provide important complementary for experimental studies. Especially, plenty of evidences have demonstrated that lncRNAs plays important roles in various kinds of human cancers^36^,^37^,^38^,^39^,^40^,^41^. Therefore, case studies about three kinds of important cancers were implemented to show the predictive performance of HGLDA. Predictive results were confirmed based on recent experimental literatures.

As the second leading cause of female cancer death, breast cancer comprises 22% of all cancers in women^67^,^68^. Breast cancer is caused because of multiple molecular alterations and traditionally diagnosed based on histopathological features such as tumor size, grade and lymph node status^69^. Researches showed that lncRNA plays an important role in many biological processes and is strongly associated with the formation of various cancers including breast cancer^69^,^70^. To better diagnose and treat breast cancer, it is necessary to predict breast cancer-related lncRNAs and identify lncRNA biomarkers^70^. HGLDA was implemented to prioritize candidate lncRNAs for breast cancer. As a result, seven lncRNAs with significant FDR less than 0.05 have been confirmed based on recent experimental literatures (see Table 1). For example, XIST, KCNQ1OT1 and NEAT1 are there experimentally confirmed breast cancer related lncRNAs, which have been ranked 1st, 8th, and 12th in the predicted list based on the model of HGLDA, respectively. The XIST RNA signal variability in the BRCA1 breast tumor is correlated with chromosomal genetic abnormalities, and BRCA1 breast tumors often contain cells showing multiple XIST RNA domains per nucleus^71^. KCNQ1OT1 is induced by estrogen in estrogen receptor-alpha (ERα) expressing breast cancer cells and further mediate CDKN1C repression through epigenetic repression^72^. The alternative splicing of NEAT1 may play important role in nicotine induced breast cancer development^73^ and breast cancer patients with high level of NEAT1 expression shows low survival rate^74^.

Lung cancer, which can be roughly divided into two groups: non-small cell lung cancer (80.4%) and small cell lung cancer (16.8%) considering disease patterns and treatment strategies, is the leading cause of cancer-related death worldwide in both men and women^75^,^76^. There are estimated 1.4 million deaths resulting from lung cancer each year^77^,^78^. Data show that the risk of lung cancer mortality is even greater than the combination of the next three most common cancers (colon, breast and prostate)^75^. Specially, five-year survival rate of lung cancer patients is only approximately 15%, which is much lower than other cancers types^79^,^80^. To diagnose and treat lung cancer in a better and more efficiently way, more attentions are focused on the deregulation of protein-coding genes to identify oncogenes and tumor suppressors in the last decades^75^,^81^,^82^. Recent researches have shown that lncRNAs play a critical role the development and progression of lung cancers^75^,^82^. Potential lung cancer-related lncRNAs were obtained by selecting candidate lncRNAs with FDR less than 0.05. Seven predicted lncRNAs have been confirmed by independent experimental literatures (see Table 1). According to biological experiments in several studies, it has been confirmed that MALAT1 is a non-coding RNA which plays important roles in many different cancers^47^. Specially it has been shown to be highly associated with metastasis of lung cancer^83^,^84^,^85^,^86^ and promote lung cancer cell motility by regulating motility related gene expression^87^. Therefore, it could be an important biomarker for metastasis development in lung cancer^49^. TUG is another lung cancer related lncRNA, which can be regulated by P53 to affect non-small cell lung cancer (NSCLC) cell proliferation in part by epigenetically controlling the expression of HOXB7^88^. GAS5, which can also be mediated by P53 pathway, is shown to be a tumor suppressor and down-regulated in NSCLC^89^. These three lncRNAs were all ranked in the top of prediction list for lung cancer (10th, 14th, and 41st, respectively).

As the third most common cancer in men and the second in women, colorectal cancer is one of the most common malignancies in the world and an important threat to human health^90^,^91^. Data shows that the 5.2% of men and 4.8% of women have the risk of colorectal cancer in the United States and the mortality rate caused by colorectal cancer is nearly 33% in the developed world^90^,^91^,^92^. Some critical mutations underlying the pathogenic mechanism of colorectal cancer have been confirmed^93^. Especially, mutations and dysregulations of some lncRNAs have been linked with the development and progression of colorectal cancer. Five predicted colorectal cancer-related lncRNAs have been confirmed by experimental literature (see Table 1). XIST, MALAT1, H19, and KCNQ1OT1 were ranked in the top four prediction list of colorectal cancer. As a result, recent biological experiments indicated these four lncRNAs all showed high correlation with colorectal cancer. For example, evidences show that expression level change of or DNA amplification of XIST is associated with colorectal carcinoma^94^,^95^. Also, MALAT1 plays important role in colorectal cancer development by promoting its invasion and metastasis^96^,^97^,^98^,^99^, and down-regulation of MALAT1 will inhibit colorectal invasion by attenuating Wnt/β-catenin signaling^100^. Moreover, the methylation state of H19 locus is highly related with colorectal cancer^101^,^102^,^103^,^104^,^105^, and the H19-derived microRNA also regulates colorectal cancer development^106^. Loss of imprinting of KCNQ1OT1 is considered as a useful marker for diagnosis of colorectal cancer because of its frequent occurrences in colorectal cancer samples^107^.

### lncRNA functional similarity

LFSCM was applied to all the lncRNAs investigated in this study. Therefore, pairwise functional similarity among 1114 lncRNAs has been obtained (See Supplementary Table 2).

## Discussions

Predicting potential disease-related lncRNAs by integrating various kinds of biological datasets is one of the most important and attracting topics for computational biology research, which is critical for understanding disease mechanism at the lncRNA level and disease biomarkers detection for disease diagnosis, prognosis and prevention. In this study, considering many miRNA-disease associations have been confirmed by recent biological experiments, the model of HGLDA was developed to predict potential disease-lncRNA associations on a large scale by selecting disease-lncRNA pairs which significantly share common miRNA partners. The important difference from previous computational researches about lncRNA-disease inference is that HGLDA doesn’t rely on any known lncRNA-disease associations. To validate the performance of HGLDA, LOOCV was implemented on lncRNA-disease association dataset obtained from lncRNADisease database and case studies were further implemented to three important cancers (Breast cancer, Lung Cancer, and Colorectal Cancer). Reliable performance has been obtained in the above validations. Therefore, to facilitate further biological experiment confirmation, significant lncRNA-disease pairs for all the diseases investigated in this study were publicly released. It is anticipated that HGLDA could further demonstrate its potential value for disease-lncRNA association inference and disease biomarker detection in the future.

Calculating lncRNA functional similarity could benefit lncRNA function inference and disease-related lncRNA prioritization. Therefore, based on the assumption that functionally similar lncRNAs tend to interact with functionally similar miRNAs, the model of LFSCM was further developed to quantitatively calculate lncRNA functional similarity. In this model, disease semantic similarity, miRNA-disease associations, and miRNA-lncRNA interactions were integrated on a large scale.

HGLDA obtained the reliable performance in both LOOCV and case studies about three kinds of important cancers, which could be largely attributed to the following several factors. Firstly, known experimentally verified disease-miRNA associations and lncRNA-miRNA interactions were integrated to infer the potential associations between lncRNAs and diseases. Secondly, both miRNA and lncRNA are ncRNAs, which don’t encode protein sequences. Therefore, predicting lncRNA-disease associations from miRNA-related datasets is more reasonable than previous study of integrating disease genes and gene-lncRNA co-expression relationship. More importantly, HGLDA doesn’t need the prior information of known lncRNA-disease associations, which ensure that this method could be applied to the diseases without any known related lncRNAs. Therefore, HGLDA represents a novel, effective, and important bioinformatics tool for the research of both complex diseases and lncRNAs.

Despite of the reliable performance of HGLDA, there are also some limitations in the model of HGLDA. Although HGLDA doesn’t rely on any known experimentally verified lncRNA-disease associations, its performance was not very satisfactory based on the evaluation of LOOCV and could be further improved by integrating more reliable biological datasets, such as disease semantic similarity, disease phenotypic similarity, lncRNA functional similarity, and lncRNA-related various interactions. Although the model of LFSCM can be applied to the lncRNAs without any known related diseases, it can’t be applied to those lncRNAs without any known miRNA interaction partners. Furthermore, lncRNA functional similarity was calculated based on known miRNA-disease associations and lncRNA-miRNA interactions, hence LFSCM tends to cause bias to lncRNAs with more miRNA interaction partners or/and lncRNAs with miRNA interaction partners which has been associated with more diseases. LFSCM would be further improved when more known datasets could be available and more reliable types of biological datasets could be integrated. More importantly, as what has been pointed out in the literature^108^, it is unwise to use a single disease-related lncRNA to judge cancer risks for all the persons. Therefore, I planned to construct various cancer hallmark networks to effectively evaluate cancer risks based on the lncRNA profiles of each person^108^. Finally, obtaining the tumor recurrence and metastases probability, predicting potential consequences after applying a specific drug to the patients, and identifying molecular signatures to evaluate and predict therapeutic results after cancer treatment in the framework of lncRNAs are three important problems in the personalized medicine^108^,^109^, which could be considered in the future.

## Methods

### Human miRNA-disease associations

The human miRNA-disease association dataset was downloaded from HMDD in January, 2015, which included 10368 high-quality experimentally verified human miRNA-diseases associations from 3511 papers about 572 miRNA and 378 diseases^110^. Then, duplicate associations with the different evidences were discarded and different miRNA copies were merged which produce the same mature miRNA. Finally, 5430 miRNA–disease associations were obtained, including 383 diseases and 495 miRNAs (see Supplementary Table 3).

### lncRNA–miRNA interactions

lncRNA–miRNA interaction dataset was downloaded from starBase v2.0 database in January, 2015, which provided the most comprehensive experimentally confirmed lncRNA–miRNA interactions based on large scale CLIP-Seq data^111^. After getting rid of duplicate interactions, 10112 lncRNA-miRNA interactions about 132 miRNAs and 1114 lncRNAs were obtained (see Supplementary Table 4).

### Disease-lncRNA associations

To validate the performance of HGLDA, the recent version of lncRNA-disease association dataset in the LncRNADisease database was downloaded^29^ and LOOCV was implemented based on this golden-standard dataset. For this dataset, I got rid of duplicate associations with different evidences and the lncRNA-disease associations involved with either diseases or lncRNAs which were not contained in the dataset used in this paper. As a result, 183 lncRNA-disease associations were obtained and LOOCV was implemented based on these experimentally verified high-quality associations (see Supplementary Table 5).

### HGLDA

**Figure 2:**
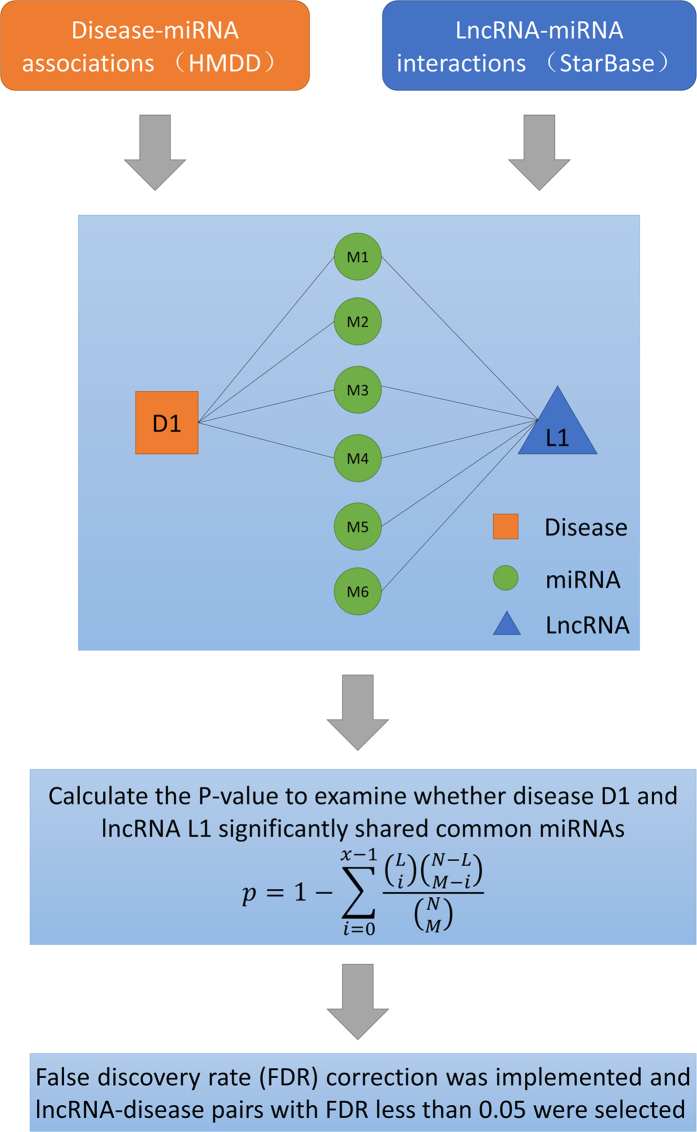
Flowchart of HGLDA, demonstrating the basic ideas of predicting potential disease-related lncRNAs by integrating miRNA-disease associations and lncRNA-miRNA interactions. Firstly, the hypergeometric distribution test was implemented for each lncRNA-disease pair by calculating the P-value to indicate whether this lncRNA and disease significantly shared common miRNAs which can interact with both of them. Then, FDR correction was implemented to all calculated P-values. Finally, those lncRNA-disease pairs with FDR less than 0.05 were selected to be potential lncRNA-disease associations.

The model of HGLDA was developed to predict potential disease-related lncRNAs (See Fig. 2). The hypergeometric distribution test was implemented for each lncRNA-disease pair by examining whether this lncRNA and disease significantly shared common miRNAs which can interact with both of them. The significance was measured by the P-value defined as follows:


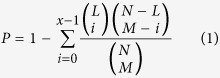


where N is the total number of miRNAs which are associated with lncRNAs or diseases, M is the number of miRNAs interacting with this given lncRNA, L is the number of miRNAs interacting with this given disease, and x is the number of miRNAs that interact with both of them, respectively. Furthermore, FDR correction was implemented to all calculated P-values and those lncRNA-disease pairs with FDR less than 0.05 were considered to be potential lncRNA-disease associations^112^.

### LFSCM

**Figure 3:**
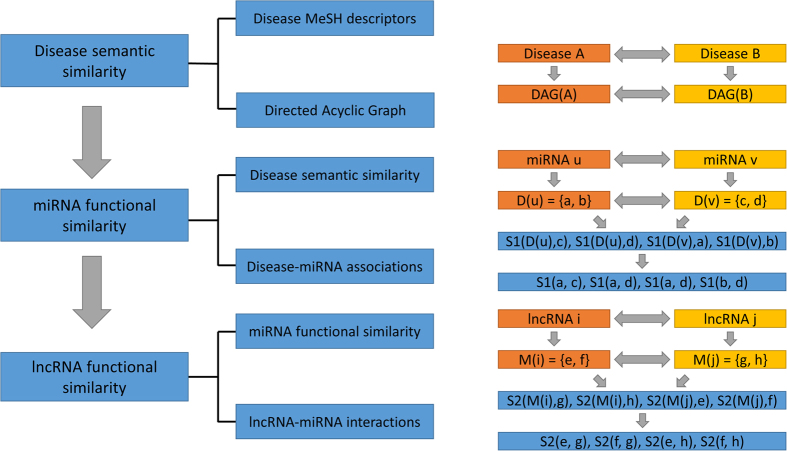
Flowchart of LFSCM, demonstrating the basic ideas of calculating lncRNA functional similarity based on disease semantic similarity, disease-miRNA associations, and lncRNA-miRNA interactions. Firstly, disease semantic similarity among all the diseases investigated in this paper was calculated based on their disease DAGs. Then, disease set associated with each miRNA was identified and the similarity among these disease sets was calculated and considered to be miRNA functional similarity. Finally, lncRNA functional similarity was calculated based on miRNA functional similarity and lncRNA-miRNA interactions.

LFSCM is composed of the following three steps (See Fig. 3): calculating disease semantic similarity based on the disease MeSH descriptors and their direct acyclic graphs (DAGs); calculating miRNA functional similarity based on disease semantic similarity and disease-miRNA associations; calculating lncRNA functional similarity based on miRNA functional similarity and lncRNA-miRNA interactions. For the disease semantic similarity calculation, the method in the literature^113^ was adopted. The semantic similarity between two diseases was calculated based on the nodes shared by their disease DAGs. The variable *S1* is denoted as disease semantic similarity matrix, in which the entity *S1(i,j)* in row *i* column *j* represents the semantic similarity between disease *i* and *j*.

For the miRNA functional similarity, the semantic similarity of their associated disease groups was measured. the similarity calculation between miRNA *u* and *v* is taken as an example to demonstrate the procedure, which consisted of three steps: obtaining all the known diseases associated with miRNA *u* and *v*, which are defined as variable *D(u)* and *D(v)* , respectively; calculating the similarity between each disease in one disease groups and the other disease groups; calculating the similarity between two disease groups as the functional similarity between miRNA *u* and *v*. In the second step, taking the similarity calculation between *D(v)* and disease *D1* in the groups of *D(u)* as an example, similarity was defined as follows:


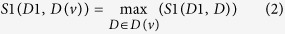


In the third step, the functional similarity between miRNA *u* and *v* was defined


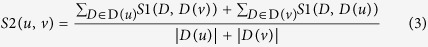


where *S2* is the miRNA functional similarity matrix and the entity *S2(i,j)* in row *i* column *j* is the functional similarity between miRNA *i* and *j*.

For the lncRNA functional similarity calculation, similar method as miRNA functional similarity calculation was adopted. Here, lncRNA *i* and *j* is take as an example. Firstly, all the miRNAs interacting with these two lncRNA as miRNA groups are defined as *M(i)* and *M(j)*, respectively. Then, the similarity between miRNA group *M(j)* and miRNA *M1* in the miRNA group *M(i)* was defined as follows:


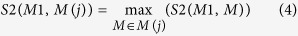


Finally, the similarity between two miRNA groups was calculated and regarded as the functional similarity between corresponding two lncRNAs.


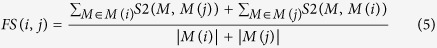


where *FS* is the lncRNA functional similarity matrix and the entity *FS(i,j)* in row *i* column *j* is the functional similarity between lncRNA *i* and *j*.

## Additional Information

**How to cite this article**: Chen, X. Predicting lncRNA-disease associations and constructing lncRNA functional similarity network based on the information of miRNA. *Sci. Rep.* **5**, 13186; doi: 10.1038/srep13186 (2015).

## Supplementary Material

Supplementary Information

Supplementary Table 1

Supplementary Table 2

Supplementary Table 3

Supplementary Table 4

Supplementary Table 5

## Figures and Tables

**Table 1:** HGLDA was applied to three kinds of important cancer (breast cancer, lung cancer, and colorectal cancer).

**Disease**	**lncRNA**	**Evidence (PMID)**
Breast cancer	MALAT1	24525122;19379481
Breast cancer	H19	16707459;14729626;12419837
Breast cancer	CDKN2B-AS1	17440112;20956613
Breast cancer	NEAT1	25417700;23825647
Breast cancer	XIST	17545591
Breast cancer	KCNQ1OT1	21304052
Breast cancer	HOTAIRM1	25296969
Lung cancer	EPB41L4A-AS1 BCYRN1	16973895;9490301
Lung cancer	MALAT1	20937273;24757675;24667321
Lung cancer	TUG1	24853421
Lung cancer	GAS5	24357161;23676682
Lung cancer	HOTAIR	25491133;24591352;24155936
Lung cancer	H19	16707459;8838103;7700644
Lung cancer	NEAT1	25010625
Colorectal cancer	XIST	17143621;22879877
Colorectal cancer	HOTAIR	24531795;21862635;24667321
Colorectal cancer	MALAT1	21503572;25446987;25031737
Colorectal cancer	KCNQ1OT1	16965397;11340379;23660942
Colorectal cancer	H19	18719115;19926638;22121898

As a result, 19 predicted lncRNA-disease pairs with significant FDR less than 0.05 have been confirmed based on recent experimental literatures.
