# Supplementary material for: Predicting lncRNA-disease associations and constructing lncRNA functional similarity network based on the information of miRNA
Source: Sci Rep. 2015 Aug 17;5:13186. doi: 10.1038/srep13186 (PMC4538606; doi:10.1038/srep13186)
Supplement: Supplementary Information [file srep13186-s1.doc]

**Predicting lncRNA-disease associations and constructing lncRNA functional similarity network based on the information of miRNA**

Xing Chen^1, 2,*^

^1^National Center for Mathematics and Interdisciplinary Sciences, Chinese Academy of Sciences, Beijing, 100190, China

^2^Academy of Mathematics and Systems Science,

Chinese Academy of Sciences, Beijing, 100190, China

*Corresponding authors

**Email**: [xingchen@amss.ac.cn](mailto:xingchen@amss.ac.cn)

**Supplementary Information**

**Supplementary Table 1.** Potential disease-lncRNA associations predicted by HGLDA for each disease were publicly released to benefit the biological experimental validation.

**Supplementary Table 2.** Pairwise functional similarity among 1114 lncRNAs investigated in this paper calculated by the model of LFSCM.

**Supplementary Table 3.** The human miRNA-disease association dataset was downloaded from HMDD in January, 2015. After getting rid of duplicate associations with the different evidences and merging different miRNA copies which produce the same mature miRNA, this dataset consists of 5430 miRNA–disease associations, including 383 diseases and 495 miRNAs.

**Supplementary Table 4.** The lncRNA–miRNA interaction dataset was downloaded from starBase v2.0 database in January, 2015. After getting rid of duplicate interactions, 10112 lncRNA-miRNA interactions about 132 miRNAs and 1114 lncRNAs were obtained.

**Supplementary Table 5.** The recent version of lncRNA-disease association dataset in the LncRNADisease database was downloaded. After getting rid of duplicate associations with different evidences and the lncRNA-disease associations involved with either diseases or lncRNAs which were not contained in the dataset used in this paper, this dataset consists of 183 lncRNA-disease associations. LOOCV was implemented based on these experimentally verified high-quality associations to validate the performance of HGLDA.
